# Runt-Related Transcription Factor 1 (RUNX1) Promotes TGF-β-Induced Renal Tubular Epithelial-to-Mesenchymal Transition (EMT) and Renal Fibrosis through the PI3K Subunit p110δ

**DOI:** 10.1016/j.ebiom.2018.04.023

**Published:** 2018-05-11

**Authors:** Tong Zhou, Maocai Luo, Wei Cai, Siyuan Zhou, Danying Feng, Chundi Xu, Hongyan Wang

**Affiliations:** a Department of Pediatrics, Ruijin Hospital, Shanghai Jiaotong University School of Medicine, China; b State Key Laboratory of Cell Biology, Key Laboratory of Systems Biology, CAS Center for Excellence in Molecular Cell Science, Innovation Center for Cell Signaling Network, Shanghai Institute of Biochemistry and Cell Biology, Chinese Academy of Sciences, University of Chinese Academy of Sciences, Shanghai, China; c Department of Surgery, Ruijin Hospital, Shanghai Jiaotong University School of Medicine, China

**Keywords:** RUNX1, EMT, Renal fibrosis, PI3K, p110δ

## Abstract

Renal fibrosis is widely considered a common mechanism leading to end-stage renal failure. Epithelial-to-mesenchymal transition (EMT) plays important roles in the pathogenesis of renal fibrosis. Runt-related transcription factor 1(RUNX1) plays a vital role in hematopoiesis via Endothelial-to-Hematopoietic Transition (EHT), a process that is conceptually similar to EMT, but its role in EMT and renal fibrosis is unclear. Here, we demonstrate that RUNX1 is overexpressed in the processes of TGF-β-induced partial EMT and renal fibrosis and that the expression level of RUNX1 is SMAD3-dependent. Knockdown of RUNX1 attenuated both TGF-β-induced phenotypic changes and the expression levels of EMT marker genes in renal tubular epithelial cells (RTECs). In addition, overexpression of RUNX1 promoted the expression of EMT marker genes in renal tubular epithelial cells. Moreover, RUNX1 promoted TGF-β-induced partial EMT by increasing transcription of the PI3K subunit p110δ, which mediated Akt activation. Specific deletion of *Runx1* in mouse RTECs attenuated renal fibrosis, which was induced by both unilateral ureteral obstruction (UUO) and folic acid (FA) treatment. These findings suggest that RUNX1 is a potential target for preventing renal fibrosis.

## Highlights

• RUNX1 is required for TGF-β induced renal tubular EMT, which increases p110δ transcription for Akt activation.
• Ablation of RUNX1 in mouse RTECs inhibits renal fibrosis induced by unilateral ureteral obstruction or folic acid.
• These findings suggest that RUNX1 might be used as a potential target to prevent renal fibrosis.

Kidney fibrosis is a critical pathologic step during the development of renal failure, while epithelial-to-mesenchymal transition (EMT) contributes to the pathogenesis of renal fibrosis. Exploring the new effectors as potential targets to inhibit renal fibrosis is currently under extensive investigation. This manuscript has identified that RUNX1 is required for TGF-β induced renal tubular EMT via increasing expression levels of the PI3K subunit p110δ and Akt activation. Importantly, ablation of *Runx1* in mouse renal tubular epithelial cells or the RUNX1 inhibitor could reduce renal fibrosis in response to unilateral ureteral obstruction or under the treatment of folic acid. These findings suggest that the RUNX1 inhibitor might be used to prevent renal fibrosis.

## 1 Introduction

Chronic kidney disease is one of the leading cause of death worldwide, is highly prevalent in many countries, and is becoming a major public health problem [1]. Renal fibrosis, which is characterized by the excessive deposition of extracellular matrix in the kidney, is the common final pathologic pathway of nearly all chronic kidney diseases and leads to end-stage renal failure [2]. Although great efforts have been put into finding the molecular and cellular regulators of kidney fibrosis in recent years, there are currently no effective therapies to prevent the onset or progression of renal fibrosis [1]. Many studies have established TGF-β as the master regulator of renal fibrosis [50]. In addition to renal fibrosis, TGF-β regulates many other biological process, such as cell apoptosis, proliferation, differentiation and immune responses; thus, directly targeting TGF-β may have adverse effects [50]. Therefore, it is imperative to explore the molecular and cellular mechanisms underlying TGF-β-induced renal fibrosis, which might provide new treatment strategies.

TGF-β is the most important inducer of epithelial-to-mesenchymal transition (EMT) in embryogenesis, fibrosis, and cancer [3], and EMT is the key mechanism underlying TGF-β-driven renal fibrosis [50]. Renal EMT is a process in which renal tubular epithelial cells (RTECs) lose epithelial cell markers, such as E-cadherin or Ksp-cadherin, and gain mesenchymal cell markers, such as N-cadherin, fibronectin, vimentin. RTECs are the major constituents of the renal parenchyma and are often the target in kidney injury [4]. Many studies have observed the phenomenon of EMT and its role in renal fibrosis [5]. Genetic ablation of key TGF-β-induced EMT targets *Tgfb2* [6], *Smad4* [7], *Snai1* [8] and *Twist1* [9], specifically in RTECs, can prevent the progression of renal fibrosis. Consistently, overexpressing Snai1 in tubular epithelial cells induces fibrosis [10]. Partial EMT, a status that RTECs do not transdifferentiate into interstitial fibroblasts but remain integrated in the tubules, could induce RTECs dysregulation of absorption, secretion, cell cycle and repair [11]. Partial EMT is one of the important mechanisms for renal fibrosis progression [8,9,11].

TGF-β-induced renal fibrosis and EMT includes both a Smad-dependent pathway, which involves the activation of Smad2/3/4, and Smad-independent pathways, including the activation of JNK, p38, ERK, and PI3K/Akt [12]. Many co-activators or co-repressors are known to interact with Smads, including the Runx family of transcription factors RUNX1, RUNX2 and RUNX3 [13]. Previous studies have shown that RUNX2 mediates the antiapoptotic effects of parathyroid hormone in proximal tubule cells [14] and that RUNX3 is involved in regulating the expression of AT1 receptor-associated proteins in renal distal convoluted tubule cells [15]. RUNX1 is critical for generating definitive hematopoietic stem cells via the Endothelial-to-Hematopoietic Transition (EHT) [16], which is conceptually similar to EMT. In addition, the role of RUNX1 in non-immune cells has recently received great attention, such as lung epithelial cells [17], gastric epithelial cells [18], colon epithelial cells [19], hepatocytes [20], and mesenchymal stem cells [21]. However, the roles of RUNX1 in TGF-β-induced EMT and renal fibrosis are still unclear.

In this study, we used a conditional knockout mouse model that specifically deleted RUNX1 in proximal tubular epithelial cells and investigated whether and how RUNX1 mediated renal fibrosis and EMT. Our results show that RUNX1 expression was enhanced both in response to TGF-β-treatment and in renal fibrosis. RUNX1 promoted TGF-β-induced partial EMT by increasing transcription of the PI3K subunit p110δ. Deletion of RUNX1 in RTECs protected the host against renal fibrosis induced by unilateral ureteral obstruction (UUO) or treatment with folic acid (FA).

## 2 Materials and Methods

### 2.1 Reagents

Antibodies against RUNX1, SLUG and N-cadherin were purchased from Santa Cruz Biotechnology (Santa Cruz, CA, USA). Antibodies against RUNX1 for IHC were from Abcam (Cambridge, MA, USA). Antibodies against SNAI1, α-SMA, Vimentin, SMAD4, p110δ, p-AKT, p-p38, p-ERK and p-SMAD3 were purchased from Cell Signaling Technology (Danvers, MA, USA). Antibodies against GAPDH, and secondary HRP-conjugated goat anti-mouse and anti-rabbit IgG were purchased from Beyotime Biotechnology (Shanghai, China). Electrochemiluminescent (ECL) reagents were purchased from Thermo Fisher Scientific (San Jose, CA, USA). Recombinant human TGF-β was purchased from PeproTech (Rocky Hill, NJ, USA). P110δ inhibitor CAL-101, PI3K inhibitor LY294002 and SMAD3 inhibitor SIS3 were purchased from Selleck Chemicals (Houston, TX, USA). Folic acid was purchased from Sigma-Aldrich (St. Louis, MO, USA). The siGENOME SMARTpool human *RUNX1* siRNA was obtained from Dharmacon (Lafayette, CO, USA). *SMAD3*, *PTEN*, *ATP1B1*, and *PIK3CD* siRNA were purchased from Santa Cruz Biotechnology (Santa Cruz, CA, USA). Lipofectamine RNAiMAX and Lipofectamine 2000 were purchased from Invitrogen (Carlsbad, CA, USA). The Dual-Glo Luciferase Assay System was purchased from Promega (Madison, WI, USA). The RNAiso reagent was obtained from TaKaRa Ltd. (Kyoto, Japan).

### 2.2 Cell Culture

HEK 293T cells (kind gifts from Dr. J. F. Chen, SIBCB) and NRK-52E cells (Cell Bank, Chinese Academy of Sciences) were maintained in DMEM containing 10% FBS, penicillin (100 units/ml), streptomycin (100 μg/ml) and 1% l-glutamine. HK-2 cells (Cell Bank, Chinese Academy of Sciences) and RPTEC/TERT1 cells (Kelei Biological Technology Co., Ltd) were maintained in DMEM/F12 containing 10% FBS, penicillin (100 units/ml), streptomycin (100 μg/ml) and 1% l-glutamine. HK-2 cells (5 × 10^4^/well) or RPTEC/TERT1 cells (5 × 10^4^/well) were seeded in 12-well plates and then stimulated with 5 ng/ml TGF-β for 24 h in the presence of SIS3 (5 μM), LY294002 (10 μM), or CAL-101 (1 μM). NRK-52E cells (1 × 10^5^/well) were seeded in 12-well plates and then stimulated with 20 ng/ml for 48 h.

### 2.3 Animal Models

*Runx1*^loxp/loxp^ mice and *γGT-Cre* mice were obtained from the Jackson Laboratory. *Runx1*^loxp/loxp^ mice are on C57BL/6 background and γGT-Cre mice are on the mixed C57BL/6 and BALB/c background. γGT-Cre mice were bred with *Runx1*^loxp/loxp^ mice to generate a specific disruption of *Runx1* in proximal tubular cells, and these mice were crossed for 5 generations to the C57BL/6 background. The littermates on the same genetic background including the WT control mice or *Runx1* cKO mice were used in the study. Tail DNA samples were genotyped with the following primer pairs:

*γGT-Cre*-F: GCCTCTTTGACTCCAGAGTTC

*γGT-Cre-*R: CAGGGTGTTATAAGCAATCCC;

*Runx1* ^loxp/loxp^-F: GCGTTCCAAGTCAGTTGTAAGCC

*Runx1* ^loxp/loxp^ -R: CTGCATTTGTCCCTTGGTTGACG.

Renal fibrosis was induced in a UUO model by cutting between two ligated points of the left ureter in mice as previously described [22], and sham operated mice underwent the same procedure except for the obstruction. Mice were injected intraperitoneally with folic acid (250 mg/kg body weight dissolved in 300 mM NaHCO_3_) for the FA model. After 7 days (UUO model) or 30 days (FA model), blood and kidneys were collected for various analyses. All animal procedures were conducted in strict accordance with institutional guidelines and were approved by the Institutional Animal Care and Use Committee of Shanghai Institute of Biochemistry and Cell Biology (IBCB0057).

### 2.4 Retroviral Transduction

To generate retroviral particles, 6 μg MigR1-RUNX1 (a gift from Dr. Shi Jingyi, Shanghai Institute of Hematology) was co-transfected with 6 μg pCL-10A1 into 293T cells by the calcium phosphate method. Then, retroviral supernatants were collected to infect HK-2 or NRK-52E cells to overexpress RUNX1, followed by FACS sorting of GFP^+^ cells. Cells that stably overexpressed RUNX1 and control cells were seeded in 12-well plates, and the cells were stimulated with 5 ng/ml or 20 ng/ml TGF-β. Cells were collected for RT-qPCR or western blot analysis after 24 h.

### 2.5 siRNA Transfection

HK-2 or RPTEC/TERT1 cells (5 × 10^4^/well) were seeded in 12-well plates. After 24 h, they were transfected with 20 nM RUNX1, SMAD3, PTEN, ATP1B1, PTEN+RUNX1 or ATP1B1 + RUNX1 siRNA and a nonspecific siRNA using the Lipofectamine RNAiMAX kit according to the manufacturer's instructions. After 36 h, HK-2 cells were stimulated with 5 ng/ml TGF-β for an additional 24 h. Cells were then prepared for RT-qPCR or western blot analysis.

### 2.6 RT-qPCR

Total RNA was extracted with RNAiso reagent, and cDNA was generated from 1 μg RNA using M-MLV reverse transcriptase (Promega, Madison, WI, USA) and then analyzed using RT-qPCR with SYBR Green Master Mix on a CFX-96 machine (Bio-Rad, CA, USA). Primer sequences [23,24,51] are listed in the Supplemental information.

### 2.7 Coimmunoprecipitation and Western Blot Analysis

HK-2 cells were stimulated with 5 ng/ml TGF-β for 1.5 h, and then the cell lysates were collected. Endogenous interactions between RUNX1 and SNAI1, SLUG or TWIST1 were detected by coimmunoprecipitation as previously described [24]. HK-2 cells were seeded in 12-well plates and stimulated with 5 ng/ml TGF-β for 6, 12, 24, 48 and 72 h, and then whole cell lysates were subjected to immunoblotting with anti-RUNX1, SNAI1, SLUG, and N-cadherin antibodies.

### 2.8 Luciferase Assay

293 T (5 × 10^5^) cells were co-transfected with 0.5 μg of pcDNA3.1-SMAD3/4 plasmid and 0.2 μg of CAGA luciferase reporter plasmid containing 1/20 Renilla luciferase plasmid with 0.5 μg of pcDNA3.1-RUNX1 or pcDNA3.1-GFP plasmid, using the Lipofectamine 2000 reagent. The cells were allowed to recover for 24 h and were then examined with a dual luciferase reporter assay system (Promega, USA).

### 2.9 Immunohistochemistry (IHC) and Masson Trichrome Staining (MTS)

Paraffin-embedded kidney sections were prepared as previously described [49] and then analyzed by immunohistochemistry staining using anti-RUNX1 antibodies according to the 2-step plus® Poly-HRP Anti-Mouse/Rabbit IgG Detection System (ZSGB-BIO, Beijing, China). Tissue sections from kidneys were subjected to MTS (Shanghai Bogoo Biotechnology. Co., Ltd, Shanghai, China) according to the standard protocol.

### 2.10 Renal Function Evaluation

Serum was obtained from mice and analyzed for levels of blood urea nitrogen (BUN) by a AU5800 series Automatic Biochemical Analyzer (Beckman Kurt, USA).

### 2.11 Statistical Analysis

Statistical analysis was performed with GraphPad Prism 5, and statistically significant differences were determined by two-tailed Student's *t*-tests or one way of analysis of variance.

## 3 Results

### 3.1 RUNX1 Expression Levels Are Increased in TGF-β-Induced EMT and Renal Fibrosis

**Fig. 1:**
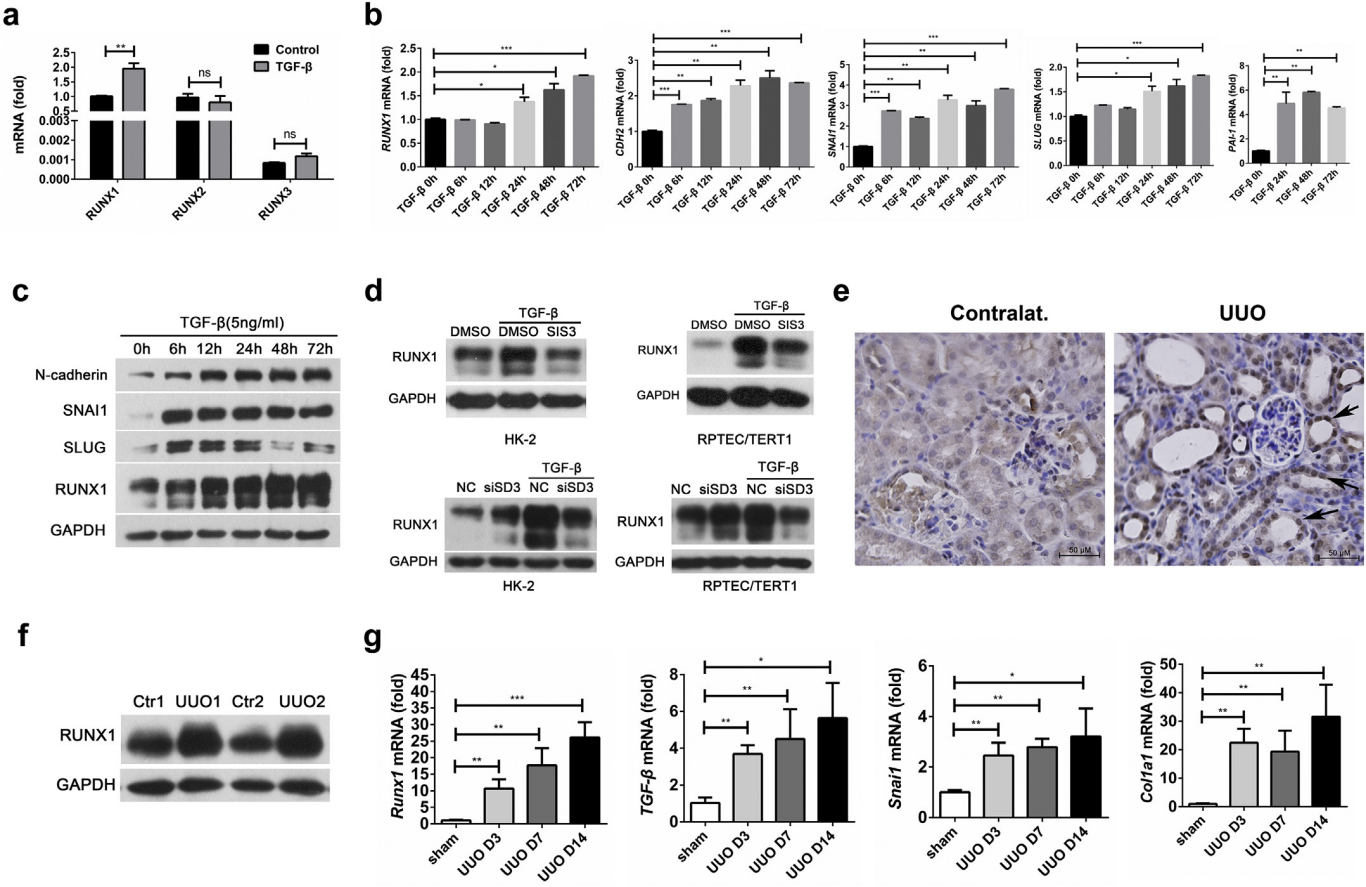
RUNX1 expression levels are increased in TGF-β-induced EMT and renal fibrosis. (a) HK-2 cells were stimulated with 5 ng/ml TGF-β for 24 h. *RUNX1*, *RUNX2* and *RUNX3* mRNA levels were detected by RT-PCR. Data are shown as the means ± SEM of three independent experiments. (b) HK-2 cells were stimulated with 5 ng/ml TGF-β for the indicated durations. Data are shown as the means ± SD of three independent experiments. (c) HK-2 cells were stimulated with 5 ng/ml TGF-β for the indicated durations to detect RUNX1, N-cadherin, SNAI1 and SLUG protein levels by immunoblotting. (d) HK-2 cells or RPTEC/TERT1 cells were transfected with SMAD3 siRNA (named siSD3) and nonspecific siRNA (named NC) or pretreated with the SMAD3 inhibitor SIS3 and control DMSO, followed by TGF-β stimulation to detect RUNX1 expression by immunoblotting. (e-g) Immunohistochemical and immunoblotting analysis of RUNX1 expression on day 7 after UUO, and *Runx1*, *Tgf*-*β*, *Snai1* and *Col1a1* mRNA levels by RT-qPCR in kidneys of UUO-induced and sham-control mice (*n* = 4). Data are shown as the mean ± SD of a representative of three independent experiments. *P < 0.05, **P < 0.01, *** *P* < 0.001.

To better understand the effects of RUNX1 in TGF-β-induced EMT and renal fibrosis, we first examined the expression of RUNX1 in human renal tubular epithelial cell line HK-2 after stimulation with TGF-β. Compared to the unchanged expression of Runx2 and Runx3, only the *RUNX1* mRNA levels were significantly increased by TGF-β treatment (Fig. 1a). In addition, we observed enhanced expression of RUNX1 and other EMT marker genes at the mRNA level, including *CDH-2*, *SNAI1* and *SLUG*, which were induced by TGF-β in a time-dependent manner (Fig. 1b). We also observed the enhanced mRNA levels of profibrogenic PAI-1 by TGF-β treatment (Fig. 1b, right panel). In addition, the enhanced expression levels of RUNX1, SNAI1, SLUG and N-cadherin were confirmed at the protein level by western blotting assays (Fig. 1c).

Next, we explored the molecular mechanisms regulating RUNX1 expression in RTECs. Because SMAD3 is the key effector in TGF-β signaling [25], we used specific siRNA or inhibitors to target SMAD3. As expected, TGF-β treatment increased RUNX1 expression at the protein level in HK-2 and another human renal proximal tubule cell line RPTEC/TERT1 cells; in contrast, knockdown and inhibition of SMAD3 prevented the upregulation of RUNX1 expression induced by TGF-β, despite a minimal increase in RUNX1 in tubular epithelial cells without TGF-β stimulation (Fig. 1d). These findings indicate that RUNX1 expression is induced in renal tubular epithelial cells upon TGF-β treatment via a SMAD3-dependent mechanism.

Because TGF-β-induced EMT plays vital roles in regulating renal fibrosis [12], we further investigated the in vivo expression of RUNX1 in kidneys from a mouse model of renal fibrosis induced by unilateral ureteral obstruction (UUO) as previously described [22]. Compared to normal kidney structure in non-obstructed contralateral kidneys of UUO mice (hereafter designated contralat. in Fig. 1e), UUO treatment obstructed kidneys via the formation of an enlarged tubular lumen. Immunohistological analysis confirmed that RUNX1 expression was enhanced in renal tubular epithelial cells, rather than in the glomerulus, in UUO-treated mice (black arrows in Fig. 1e). In agreement with these in vitro findings, *Runx1* expression at both the protein (Fig. 1e-f) and mRNA levels (Fig. 1g, left panel) was significantly increased in obstructed kidneys. In addition, we observed an upregulation of several genes related to EMT or fibrogenesis, including *TGF-β*, *Snai1* and *Col1a1*, at the mRNA level in kidneys from UUO-treated mice (Fig. 1g).

### 3.2 RUNX1 is Required for TGF-β-Induced Renal Tubular EMT

**Fig. 2:**
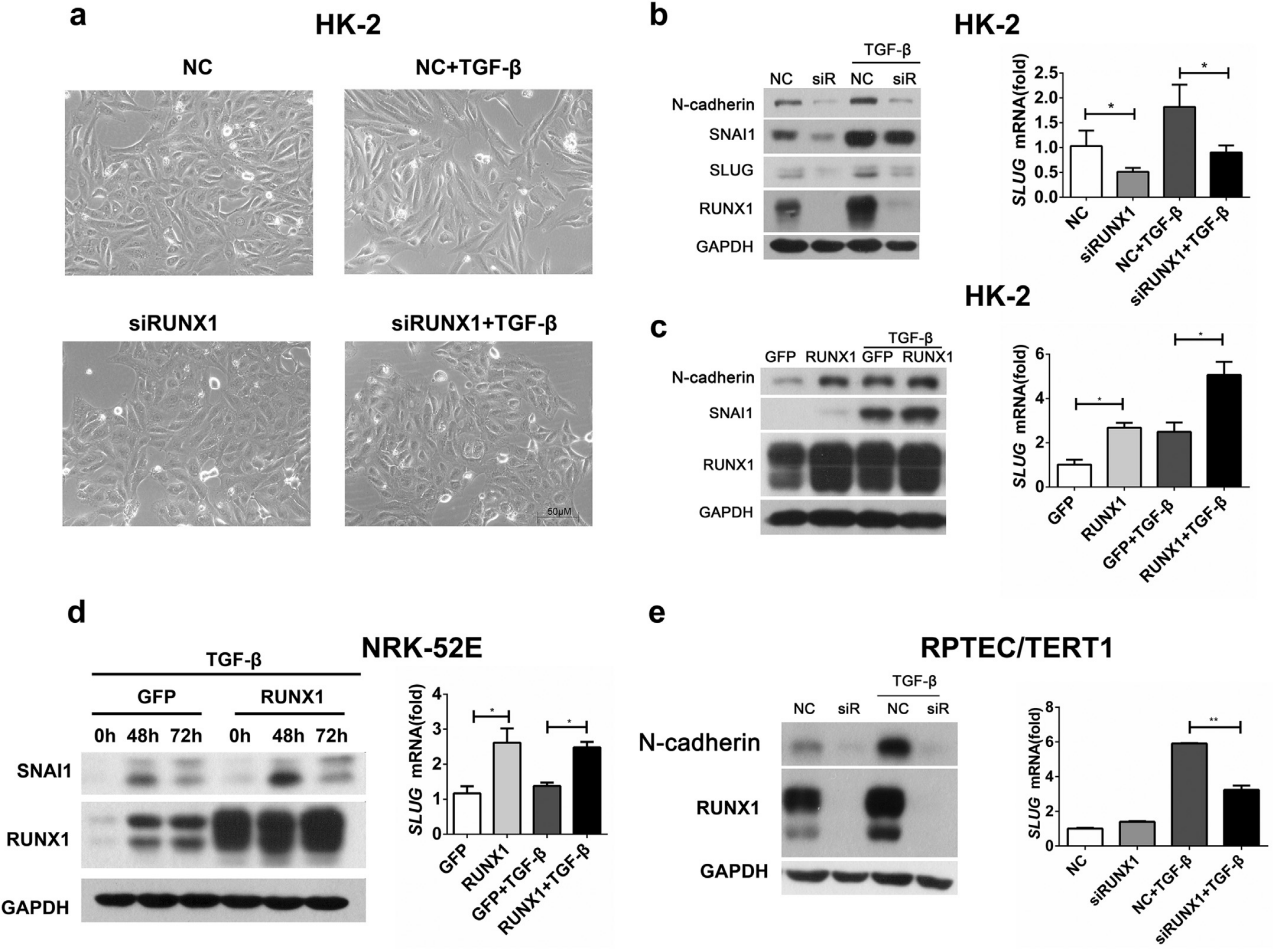
RUNX1 is required for TGF-β-induced renal tubular EMT. (A, B) HK-2 cells were transfected with RUNX1 siRNA or control siRNA followed by 5 ng/ml TGF-β stimulation for 24 h. Morphological changes of HK-2 cells (a) and RUNX1, N-cadherin, SNAI1 and SLUG expression levels were detected by immunoblotting or RT-qPCR (b). Data are shown as the means ± SEM of three independent experiments. (c, d) HK-2 cells (c) or NRK-52E cells (d) were stably transfected to overexpress RUNX1 or GFP as a control and were then stimulated with 5 ng/ml TGF-β for 24 h. RUNX1, N-cadherin and SNAI1 expression were detected at the protein level by immunoblotting, or Slug at the mRNA level by RT-qPCR. Data are shown as the means ± SEM of three independent experiments. (e) RPTEC/TERT1 cells were transfected with RUNX1 siRNA or control siRNA followed by 5 ng/ml TGF-β stimulation for 24 h. RUNX1 and N-cadherin protein or Slug mRNA expression levels were detected. Data are shown as the mean ± SD of a representative of three independent experiments. *P < 0.05, **P < 0.01.

To investigate the role of RUNX1 in EMT, we used specific siRNA to knock down RUNX1 in HK-2 cells. Light microscopy revealed that TGF-β induced morphological changes in HK-2 cells from a cobblestone-like appearance to an elongated fibroblast-like shape. In contrast, this effect was abolished by the siRNA-mediated knock-down of RUNX1 expression (termed siRUNX1) in HK-2 cells (Fig. 2a). Western blotting analysis confirmed the knock-down efficiency of RUNX1 expression by siRNA (Fig. 2b, left panel). Furthermore, in response to TGF-β stimulation, siRUNX1-transfected HK-2 cells had profoundly diminished expression of EMT marker genes at the protein level, including N-cadherin, SNAI1 and SLUG, compared with controls (Fig. 2b, left panel). In addition, siRUNX1-transfected HK-2 cells decreased *SLUG* expression at the mRNA level (Fig. 2b, right panel).

To further confirm that RUNX1 promotes renal tubular EMT, we overexpressed *RUNX1* in HK-2 cells. The overexpression levels of RUNX1 in HK-2 cells were evaluated by western blotting analysis (Fig. 2c, left panel). Overexpression of RUNX1 indeed enhanced the expression of N-cadherin and SNAI1 at the protein level (Fig. 2c, left panel) and increased *SLUG* expression at the mRNA level, compared with controls (Fig. 2c, right panel).

We next overexpressed RUNX1 in the rat renal tubular epithelial cell line NRK-52E and confirmed that the overexpression of RUNX1 increased the expression of EMT marker genes, including SNAI1 at the protein level (Fig. 2d, left panel) and SLUG at the mRNA level (Fig. 2d, right panel). We also used siRNA to knock down RUNX1 in the human tubular epithelial cell line RPTEC/TERT1, and the results showed that knockdown of RUNX1 reduced N-cadherin expression at the protein level (Fig. 2e, left panel) and SLUG at the mRNA level (Fig. 2e, right panel). Thus, we used overexpression or knockdown strategies in different cells to demonstrate that RUNX1 promotes TGF-β-induced renal tubular EMT.

### 3.3 RUNX1 Promotes EMT Via p110δ-Mediated Akt Activation

**Fig. 3:**
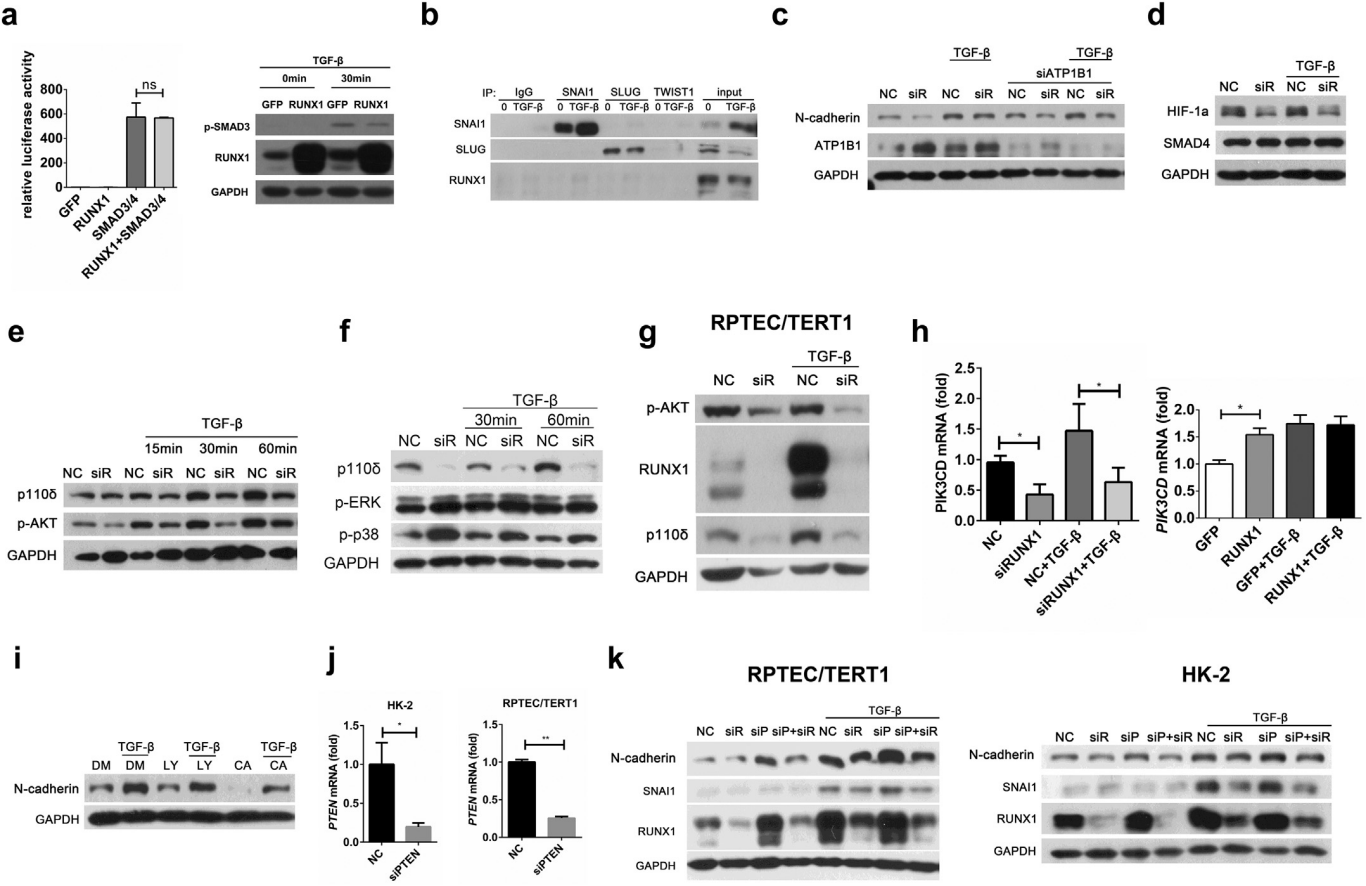
RUNX1 promotes EMT via p110δ-mediated Akt activation. (a, left panel) Plasmids for expressing GFP or RUNX1, together with plasmids for expressing SMAD3/4, were transfected into 293T cells in the presence of the CAGA luciferase reporter plasmid and the Renilla luciferase plasmid. After 24 h, cell lysates were prepared and analyzed by a dual luciferase reporter assay. Data are shown as the mean ± SD of a representative of three independent experiments. (a, right panel) NRK-52E cells were transfected with plasmids expressing RUNX1 or GFP, then stimulated with 20 ng/ml TGF-β for 30 min. RUNX1 and p-SMAD3 expression levels were detected by immunoblotting. (b) HK-2 cell lysates were immunoprecipitated with anti-SNAI1, SLUG or TWIST1 antibodies to detect endogenous interactions with RUNX1 by immunoblotting using anti-RUNX1 antibodies. (c-f) HK-2 cells were transfected with control siRNA, RUNX1 siRNA, or ATP1B1 siRNA as indicated, followed by stimulation with 5 ng/ml TGF-β for 24 h. Expression levels of N-cadherin and ATP1B1 (c) or HIF-1α and SMAD4 (d) or p110δ and phosphorylated AKT (e) or p110δ and phosphorylated ERK/p38 (f) were detected by immunoblotting. (g) RPTEC/TERT1 cells were transfected with RUNX1 siRNA or control siRNA, then stimulated with 5 ng/ml TGF-β for 24 h. p110δ, RUNX1 and Akt phosphorylation levels were detected by immunoblotting. (h) RUNX1 was knocked down or overexpressed in HK-2 cells to measure *PIK3CD* mRNA levels by RT-qPCR. Data are shown as the means ± SEM of three independent experiments. (i) HK-2 cells were stimulated with 5 ng/ml TGF-β for 24 h in the absence or presence of the PI3K inhibitor LY294002 (10 μM, named LY) or the p110δ inhibitor CAL-101 (1 μM, named CA). N-cadherin expression was detected by immunoblotting. (j, k) HK-2 or RPTEC/TERT1 cells were transfected with the control siRNA (named NC), RUNX1 siRNA (named siR), PTEN siRNA (named siP), then stimulated by 5 ng/ml TGF-β for 24 h. The mRNA levels of PTEN were detected by RT-qPCR (j). N-cadherin, RUNX1 and SNAI1 protein levels were detected by immunoblotting (k). Data are shown as the mean ± SD from three independent experiments. *P < 0.05, **P < 0.01.

SMAD3 plays a critical role in TGF-β-induced EMT, and previous reports have demonstrated a direct interaction between SMADs and RUNX1 [13]. We therefore examined the effect of RUNX1 on SMAD3 activation using a dual-luciferase reporter driven by Smad-binding CAGA elements. Unexpectedly, the co-expression of RUNX1 with SMAD3 in 293 T cells did not further increase CAGA reporter readings compared with 293 T cells transfected with SMAD3 alone (Fig. 3a, left panel). In addition, we found that overexpression of RUNX1 did not profoundly affected the phosphorylation levels of SMAD3 (Fig. 3a, right panel). We then investigated whether RUNX1 could interact with key effectors for EMT, including SNAI1, SLUG and TWIST1, by immunoprecipitation assays. However, we did not detect any interactions between RUNX1 and these EMT regulators (Fig. 3b).

Recent studies have reported that loss of ATP1B1, a Na/K-ATPase subunit, can promote renal EMT [26]. Since we observed that knockdown of RUNX1 in HK-2 cells promoted expression of ATP1B1 in the absence or presence of TGF-β treatment (Fig. 3c, middle lane), we next asked whether ATP1B1 could functionally cooperate with RUNX1 in EMT. Profound knockdown efficiency of ATP1B1 in HK-2 cells was confirmed by western blotting analysis (Fig. 3c, upper lane). Unexpectedly, knockdown of ATP1B1 did not reverse the reduced N-cadherin expression in siRUNX1 knock-down cells, with or without TGF-β treatment (Fig. 3c, upper lane).

Previous studies have reported that RUNX1 interacts with HIF-1α [27] and that HIF-1α promotes renal fibrosis via EMT [28]. We next asked whether RUNX1 affected HIF-1α expression in response to TGF-β treatment. Although knockdown of RUNX1 reduced HIF-1α expression at the protein level in resting HK-2 cells, TGF-β treatment did not affect HIF-1α expression in control HK-2 cells or RUNX1 siRNA-treated HK-2 cells (Fig. 3d). Together, these results show that RUNX1 might not depend on SMAD3, ATP1B1 or HIF-1α to promote TGF-β-induced renal tubular EMT.

TGF-β signaling may also regulate EMT via a non-SMAD dependent pathway [12,50]. We therefore asked whether RUNX1 affected the activation of MAPKs and Akt by TGF-β. The results showed that RUNX1 knockdown decreased the Akt phosphorylation levels (Fig. 3e) but not the phosphorylation levels of ERK (Fig. 3F). Although the phosphorylation levels of p38 were increased in HK-2 cells with RUNX1 knock down, as the role of p38 in promoting EMT, it could not explain the molecular mechanism of RUNX1 promoting EMT (Fig. 3f). Interestingly, we also found that siRNA-mediated knockdown of RUNX1 in HK-2 cells significantly reduced PI3K subunit p110δ expression, encoded by the gene *PIK3CD*, in the presence or absence of TGF-β treatment (Fig. 3e-f). This effect was also confirmed in RPTEC/TERT1 cells (Fig. 3g). In addition, RUNX1 knockdown or overexpression modulated p110δ expression at the mRNA level (Fig. 3h). In agreement with these findings, both the PI3K inhibitor LY294002 (named LY in Fig. 3i) and the p110δ inhibitor CAL-101 (named CA in Fig. 3i) blocked the enhancement of N-cadherin expression in response to TGF-β treatment.

Because loss of phosphatase and tensin homolog deleted on chromosome ten (PTEN) may enhance Akt activation and promote EMT [29], we next examined the effect of RUNX1 on the PTEN/Akt pathway. We confirmed the knockdown efficiency of PTEN by the specific siRNA in HK-2 and RPTEC/TERT1 cells (Fig. 3j). As expected, siRNA-mediated knockdown of PTEN (named siP in Fig. 3k) enhanced TGF-β-induced expression levels of N-cadherin and SNAI1. Co-transfection of siRUNX1 and siPTEN (named siP+siR in Fig. 3k) attenuated the enhanced expression of N-cadherin and SNAI1, when compared to HK-2 and RPTEC/TERT1 cells transfected with siPTEN alone (Fig. 3k). These results indicate that RUNX1 promotes EMT by upregulating p110δ expression and AKT activation.

### 3.4 Tubule-Specific Deletion of *Runx1* Ameliorates UUO or FA-Induced Renal Fibrosis

To further explore the role of RUNX1 in vivo, we generated conditional knockout mice with tubular epithelial cell-specific ablation of *Runx1*. Mice with the γGT-Cre locus, which express Cre recombinase uniformly in proximal tubular epithelial cells [30], were bred with homozygous *Runx1*-floxed mice. This could generate the mouse model with a specific disruption of *Runx1* in proximal tubular cells (i.e. *γGT-Cre*^*+*^*Runx1*^fl/fl^ or *γGT-Cre*^*+*^*Runx1*^fl/+^ mice, hereafter named *Runx1*^cKO^ mice). *Runx1*^cKO^ mice were fertile and showed no phenotypic abnormalities, compared to the control mice (*γGT-Cre*^*−*^*Runx1*^fl/fl^, hereafter designated WT mice). The littermates of the control mice or *Runx1*^cKO^ mice were subjected to UUO induction.

**Fig. 4:**
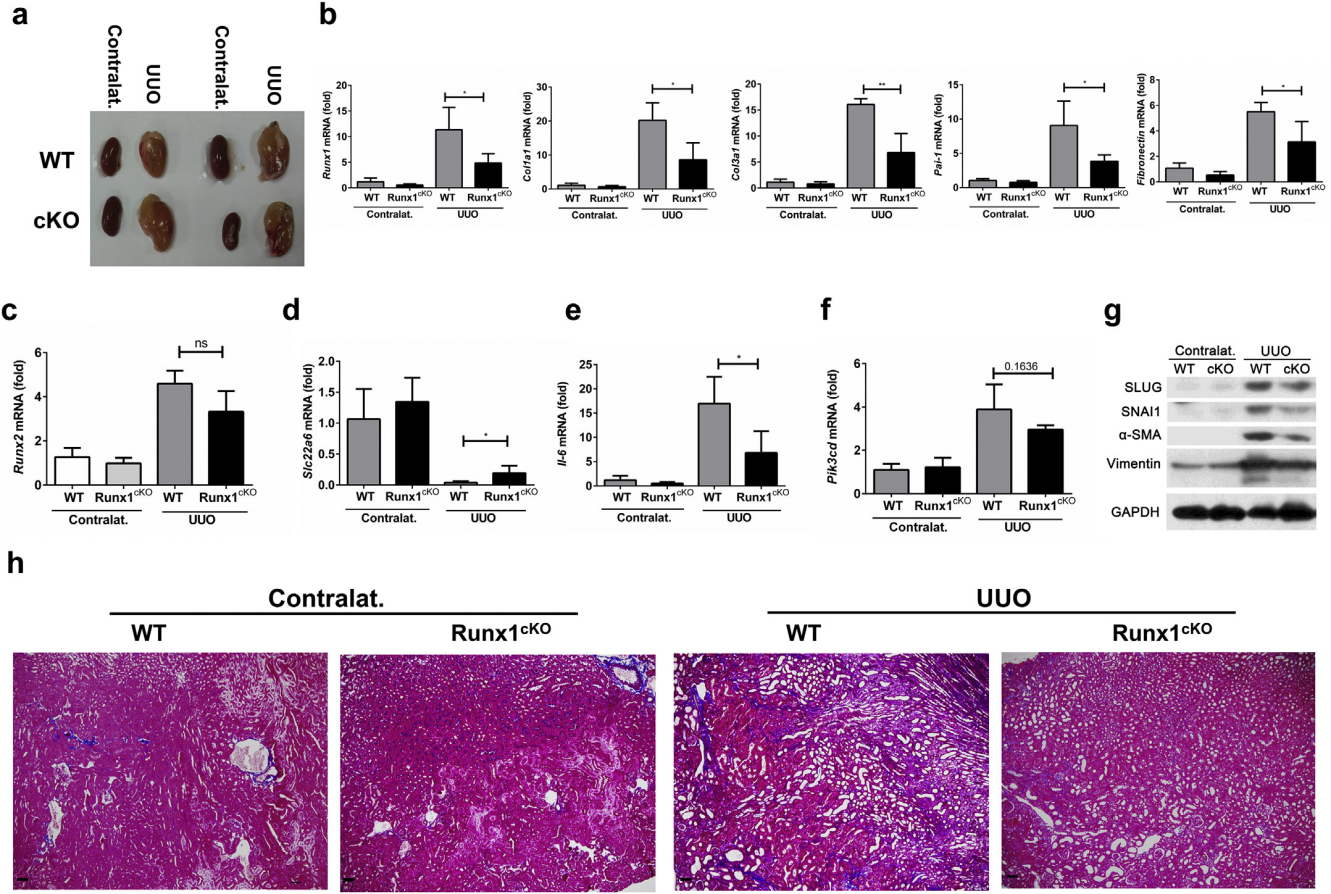
Tubule-specific deletion of *Runx1* ameliorates UUO-induced renal fibrosis. (a) Morphological changes of kidneys in control or UUO-induced Runx1^cKO^ and WT mice. (b-e) mRNA levels of *Runx1*, *Col1a1*, *Col3a1*, *Fibronectin* and *Pai-1* (b) or *Runx2* (c) or *Slc22a6* (d), or *Il-6* (e), or *Pik3cd* (f) were detected by RT-qPCR in kidneys of control and UUO-induced Runx1^cKO^ and WT mice (n = 4). (g) Immunoblotting analysis showed expression levels of SNAI1, SLUG, VIMETIN and α-SMA in kidneys of control or UUO-induced Runx1^cKO^ and WT mice. (h) Representative images of MTS staining of kidneys from the indicated groups. Black bar: 50 μm. Data are shown as mean ± SD of a representative of three independent experiments. *P < 0.05, **P < 0.01, NS, not significant.

After UUO induction, kidneys were enlarged and swollen, and there were no obvious differences in the appearance or morphology of kidneys between *Runx1*^cKO^ and WT mice (Fig. 4a). UUO treatment induced renal destruction in WT mice (Fig. 1e), which significantly enhanced mRNA expression levels of *Runx1* and profibrogenic genes including *Col1a1*, *Col3a1*, *Pai-1* and *Fibronectin* (Fig. 1e & Fig. 4b). In contrast, kidneys of *Runx1*^cKO^ mice had significantly decreased mRNA expression levels of *Runx1, Col1a1*, *Col3a1*, *Pai-1* and *Fibronectin* after UUO induction when compared to those in WT mice (Fig. 4b). Two independent groups previously reported the contradictory findings of Runx2 in renal fibrosis [14,31]. We therefore examined whether loss of RUNX1 could affect RUNX2 expression. Deletion of *Runx1* in RTECs did not substantially affect the mRNA level of *Runx2* mRNA in the UUO-induced renal fibrosis model (Fig. 4c). In addition, we checked the expression level of SLC22A6, an organic anion transporter that is involved in regulating toxicant metabolism, in kidneys from these UUO mouse models. Indeed, UUO induction significantly reduced SLC22A6 expression at the mRNA level compared to untreated healthy kidneys (Fig. 4d), and the genetic ablation of *Runx1* in kidneys partially rescued SLC22A6 expression (Fig. 4d), which indicated that RUNX1 ablation improved tubular health.

In agreement with the improvement in kidney injury and fibrosis of *Runx1*^cKO^ mice after UUO induction, mRNA levels of the inflammatory cytokine *IL-6* were also decreased (Fig. 4e). We further found that the *p110δ* mRNA levels tended to be decreased in kidneys of *Runx1*^cKO^ mice after UUO induction (Fig. 4f), although the difference between WT mice and *Runx1*^cKO^ mice was not statistically significant. Nevertheless, the expression of EMT markers at the protein level, including SNAI1, SLUG, VIMENTIN and α-SMA was significantly decreased in kidneys of UUO-induced *Runx1*^cKO^ mice compared to those in WT mice (Fig. 4g). Consistently, Masson trichrome staining (MTS) revealed a reduction of interstitial fibrosis in kidneys of *Runx1*^cKO^ mice compared to WT mice after UUO induction (Fig. 4h).

**Fig. 5:**
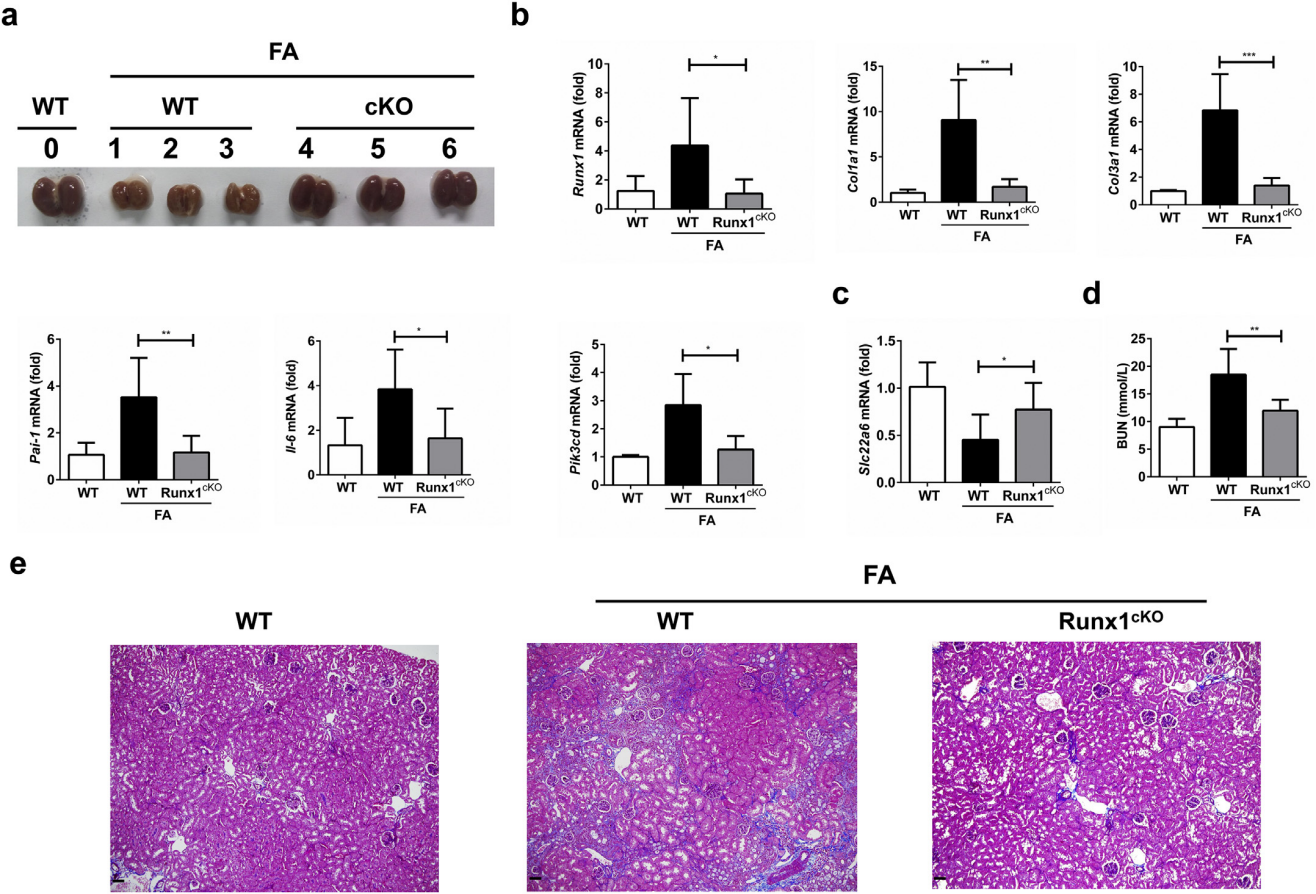
Tubule-specific deletion of *Runx1* ameliorates FA-induced renal fibrosis. (a) Morphological changes of kidneys in the control or FA-treated Runx1^cKO^ and WT mice. (b, c). The mRNA levels of *Runx1*, *Col1a1*, *Col3a1*, *Pai-1*, *Il-6* and *Pik3cd* (b), or *Slc22a6* (c) in kidneys from the control (*n* = 2) or FA-treated Runx1^cKO^ (*n* = 6) and WT mice (*n* = 9). (d) Blood urea nitrogen (BUN) levels were detected in the control or FA-treated Runx1^cKO^ and WT mice. (e) Representative images of MTS staining of kidneys from the indicated groups. Black bar: 50 μm. Data are shown as mean ± SD of a representative of three independent experiments. *P < 0.05, **P < 0.01, ***P < 0.001.

To further determine the role of RUNX1 in renal fibrosis, we utilized another renal fibrosis model induced by folic acid (FA). As shown in Fig. 5a, sizes of kidneys in WT mice were profoundly reduced after FA induction, whereas genetic ablation of *Runx1* in kidneys almost completely reversed the FA-induced changes in renal morphology. The expression levels of profibrogenic genes, including *Col1a1*, *Col3a1*, and *Pai-1,* were significantly reduced in FA-treated kidneys of *Runx1*^cKO^ mice compared to those in WT mice (Fig. 5b). The mRNA levels of *IL-6* and *p110δ* were also decreased in FA-treated kidneys of *Runx1*^cKO^ mice (Fig. 5b). Like in the UUO-induced kidney fibrosis model, ablation of RUNX1 in kidneys partially rescued the loss of SLC22A6 expression in FA-treated kidneys (Fig. 5c). After the treatment with FA, Runx1^cKO^ mice showed better renal function compared to that of WT mice (Fig. 5d). Masson trichrome staining (MTS) also confirmed that renal fibrosis was inhibited in kidneys of FA-treated *Runx1*^cKO^ mice compared to that of FA-treated WT mice (Fig. 5e). These results, taken together, suggest that RUNX1 is required for the progression of renal fibrosis.

## 4 Discussion

In the current study, we have demonstrated that expression of RUNX1, but not RUNX2 or RUNX3, was induced in the process of TGF-β-induced EMT in a SMAD3-dependent manner and in UUO-induced renal fibrosis in vivo. RUNX1 is mainly localized in RTECs in UUO-induced renal fibrosis. We used knockdown, overexpression and conditional KO mouse models to further demonstrate that RUNX1 is required for TGF-β-induced EMT and renal fibrosis in vivo. RUNX1 enhanced the expression of EMT markers including N-cadherin, SNAI1 and SLUG. Previous studies focused on the role of RUNX1 in immune cells [24,32,33], and this study has uncovered the role of RUNX1 in non-immune cells.

A recent study suggests that RUNX1 promotes the EMT process during mesendodermal differentiation via controlling TGF-β2 expression [34]. In addition, there are inconsistent or contradictory findings about whether RUNX1 promotes or inhibits EMT in breast cancer cells and epithelial ovarian carcinoma [[35], [36], [37], [38]]. Our study has identified that RUNX1 promotes renal tubular EMT and renal fibrosis. However, we did not find that RUNX1 could regulate the expression of TGF-β2 in HK-2 cells (data not shown).

Although SMAD3 is critical for TGF-β-induced EMT [25] and may interact with RUNX1 [52], we did not find that RUNX1 affected the activity of SMAD3 in dual luciferase reporter assays or the phosphorylation levels of SMAD3 in NRK-52E cells. However, our results did show that TGF-β-induced RUNX1 expression is SMAD3-dependent, which may indicate that SMAD3 promotes EMT, at least partially, via RUNX1. Although RUNX2 has been reported to interact with canonical EMT regulators [39], we excluded the possibility that RUNX1 interacts with SNAI1, SLUG or TWIST1. ATP1B1 and HIF-1α are also important in the processes of EMT and renal fibrosis [9,26,28]. Although RUNX1 may regulate ATP1B1 and HIF-1α expression, we suggest that RUNX1-induced EMT might be independent of ATP1B1 and HIF-1α because the knockdown of ATP1B1 did not reverse the EMT phenotype induced by RUNX1 and TGF-β treatment did not affect HIF-1α expression when comparing control and RUNX1 knock-down cells. We also found that RUNX1 did not affect TGF-β-mediated activation of MAPKs including ERK, and p38. After excluding the above possibility, we ultimately found that RUNX1 promotes EMT by enhancing the TGF-β-induced expression of the PI3K subunit p110δ and the phosphorylation of AKT. Our results are consistent with a previous study showing that RUNX1 directly controls the transcription of p110δ and activation of Akt in acute megakaryocytic leukemia [40]. Because the PI3K-Akt pathway controls the protein stability of SNAI1 [41] and HIF-1α [42], it may explain the observation that loss of RUNX1 only affects expression of EMT markers, including SNAIL and N-cadherin, at the protein level.

EMT has been suggested as one of the key mechanisms of TGF-β-induced fibrosis [12,50]. We found that RUNX1 promotes renal tubular EMT and that the deletion of RUNX1 specifically in RTECs significantly attenuated renal fibrosis. RUNX2 is another RUNX family member, and there is some discrepancy about the role of RUNX2 in EMT. One study used full RUNX2 KO mice and demonstrated that RUNX2 prevents renal fibrosis by inhibiting EMT and that RUNX2 expression is decreased in UUO kidneys [31]. This is contradictory to another study which reported that RUNX2 was overexpressed in UUO kidneys [14]. In addition, several studies have demonstrated that RUNX2 promotes EMT in other systems [[43], [44], [45]]. Therefore, a conditional RUNX2 knockout mouse model is needed to resolve this discrepancy. Our results show that only RUNX1, but not RUNX2 or RUNX3, is induced in renal epithelial cells by TGF-β treatment. This finding might indicate that RUNX1 plays a more important role in renal fibrosis. In agreement with previous studies showing that the inhibition of EMT prevents the loss of RTEC transporters and inhibits inflammation [8,9], deletion of RUNX1 promoted SLC22A6 expression and inhibited IL-6 expression in renal fibrosis. Interestingly, several studies have suggested that RUNX1 regulates the expression of another transporter, SLC22A4, in rheumatoid arthritis [[46], [47], [48]]. Consistent with the in vitro results, conditional KO of RUNX1 in renal tubular epithelial cells also reduced p110δ expression. This finding suggests that RUNX1 promotes renal EMT and fibrosis at least partially via p110δ-mediated Akt activation. In summary, our findings demonstrate the importance of RUNX1 in EMT and renal fibrosis, and we propose that RUNX1 might be used as a new potential therapeutic target for renal fibrosis.

## Appendix A Supplementary Data

Supplementary material Image 1
