# Supplementary material for: Runt-Related Transcription Factor 1 (RUNX1) Promotes TGF-β-Induced Renal Tubular Epithelial-to-Mesenchymal Transition (EMT) and Renal Fibrosis through the PI3K Subunit p110δ
Source: eBioMedicine. 2018 May 11;31:217–25. doi: 10.1016/j.ebiom.2018.04.023 (PMC6013935; doi:10.1016/j.ebiom.2018.04.023)

Supplementary Table 1. The primers used for Real-time qPCR.

Gene	Forward primer	Reverse primer	products size (bp)
Mouse *Tgf-β*	CTCCCGTGGCTTCTAGTGC	GCCTTAGTTTGGACAGGATCTG	133
Mouse *Snail*	ATTCTCCTGCTCCCACTGC	GACTCTTGGTGCTTGTGGAG	150
Mouse *Col1a1*	ATCTCCTGGTGCTGATGGAC	ACCTTGTTTGCCAGGTTCAC	154
Mouse *Col3a1*	ACGTAGATGAATTGGGATGCAG	GGGTTGGGGCAGTCTAGTG	154
Mouse *Pai-1*	TTCAGCCCTTGCTTGCCTC	ACACTTTTACTCCGAAGTCGGT	116
Mouse *Fibronectin*	CGAGGTGACAGAGACCACAA	CTGGAGTCAAGCCAGACACA	149
Mouse *Slc22a6*	CTGATGGCTTCCCACAACAC	GTCCTTGCTTGTCCAGGGG	133
Mouse *Il-6*	TGTATGAACAACGATGATGCACTT	ACTCTGGCTTTGTCTTTCTTGTTATCT	197
Mouse *Runx1*	TGGCAGGCAACGATGAAAAC	CGCTCGGAAAAGGACAAACTC	274
Mouse *Runx2*	TTCAACGATCTGAGATTTGTGGG	GGATGAGGAATGCGCCCTA	221
Mouse *β-actin*	CAGCTGAGAGGGAAATCGTG	CGTTGCCAATAGTGATGACC	150
Human *RUNX1*	TGAGCTGAGAAATGCTACCGC	ACTTCGACCGACAAACCTGAG	76
Human SLUG	CGAACTGGACACACATACAGTG	CTGAGGATCTCTGGTTGTGGT	87
Human *PIK3CD*	TCAACTCACAGATCAGCCTCC	CGCGAAAGTCGTTCACTTCT	84
Human PAI-1	AGTGGACTTTTCAGAGGTGGA	GCCGTTGAAGTAGAGGGCATT	151
Human GAPDH	CCCTCAACGACCACTTTGTCA	TTCCTCTTGTGCTCTTGCTGG	144

Supplementary Figure 1. Model: the regulatory role of RUNX1 in TGF-β-induced EMT.

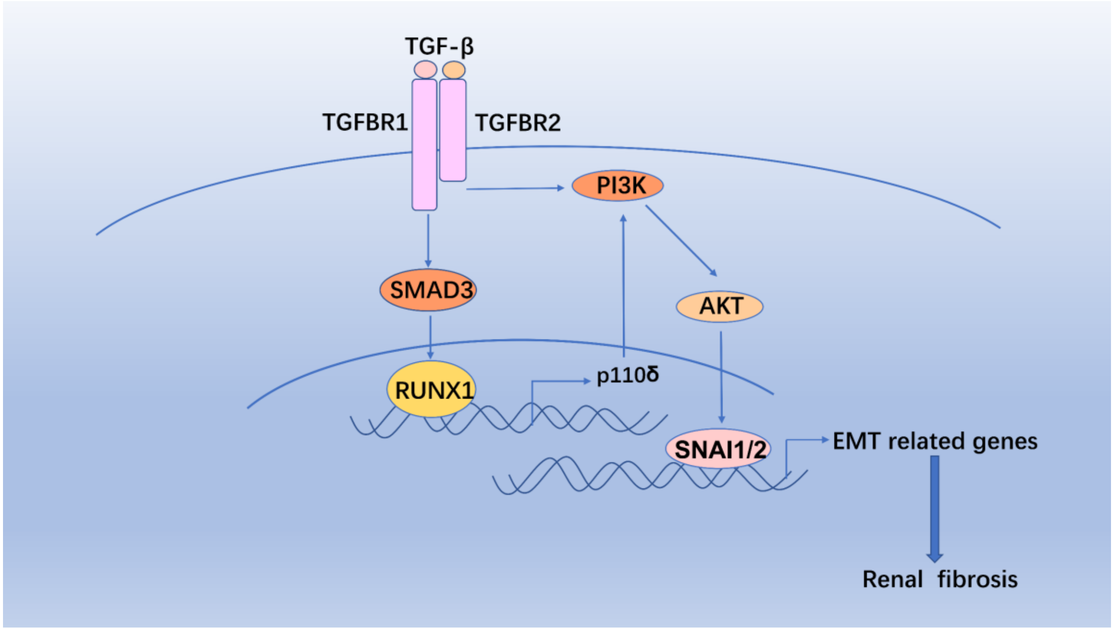

Supplement: Supplementary file 1 — Supplementary material [file mmc1.pdf]
